# Effects of Apocynin on Heart Muscle Oxidative Stress of Rats with Experimental Diabetes: Implications for Mitochondria

**DOI:** 10.3390/antiox10030335

**Published:** 2021-02-24

**Authors:** Estefanía Bravo-Sánchez, Donovan Peña-Montes, Sarai Sánchez-Duarte, Alfredo Saavedra-Molina, Elizabeth Sánchez-Duarte, Rocío Montoya-Pérez

**Affiliations:** 1 Instituto de Investigaciones Químico-Biológicas, Universidad Michoacana de San Nicolás de Hidalgo, Francisco J. Múgica S/N, Col. Felicitas del Río, Morelia 58030, Michoacán, Mexico; 1541910b@umich.mx (E.B.-S.); 0618853j@umich.mx (D.P.-M.); 1315649c@umich.mx (S.S.-D.); saavedra@umich.mx (A.S.-M.); 2 Departamento de Ciencias Aplicadas al Trabajo, Universidad de Guanajuato Campus León, Eugenio Garza Sada 572, Lomas del Campestre Sección 2, León 37150, Guanajuato, Mexico

**Keywords:** antioxidant, apocynin, diabetes, heart mitochondria, oxidative stress

## Abstract

Diabetes mellitus (DM) constitutes one of the public health problems today. It is characterized by hyperglycemia through a defect in the β-cells function and/or decreased insulin sensitivity. Apocynin has been tasted acting directly as an NADPH oxidase inhibitor and reactive oxygen species (ROS) scavenger, exhibiting beneficial effects against diabetic complications. Hence, the present study’s goal was to dissect the possible mechanisms by which apocynin could mediate its cardioprotective effect against DM-induced oxidative stress. Male Wistar rats were assigned into 4 groups: Control (C), control + apocynin (C+A), diabetes (D), diabetes + apocynin (D+A). DM was induced with streptozotocin. Apocynin treatment (3 mg/kg/day) was applied for 5 weeks. Treatment significantly decreased blood glucose levels and insulin resistance in diabetic rats. In cardiac tissue, ROS levels were higher, and catalase enzyme activity was reduced in the D group compared to the C group; the apocynin treatment significantly attenuated these responses. In heart mitochondria, Complexes I and II of the electron transport chain (ETC) were significantly enhanced in the D+A group. Total glutathione, the level of reduced glutathione (GSH) and the GSH/ oxidized glutathione (GSSG) ratio were increased in the D+A group. Superoxide dismutase (SOD) and the glutathione peroxidase (GSH-Px) activities were without change. Apocynin enhances glucose uptake and insulin sensitivity, preserving the antioxidant defense and mitochondrial function.

## 1. Introduction

Diabetes mellitus (DM) is considered an epidemic disease and is one of the fastest-growing challenges of the 21st century. The continued increase is mainly due to progressive urbanization, aging, rising obesity levels, unhealthy diets, tobacco use, and generalized physical inactivity [1]. DM can cause numerous health-weakening complications, decrease quality of life, and cause early death. It is associated with vascular and heart diseases, such as high blood pressure, coronary artery disease, and heart failure, responsible for increased morbidity and mortality [2,3]. The set of cardiovascular diseases causes about 80% of the deaths of diabetic patients. In turn, it has been linked to a broad spectrum of cardiovascular disorders, leading to atherogenesis, endothelial dysfunction, inflammation, vascular remodeling, and oxidative stress [4,5]. The increase in the production of reactive oxygen species (ROS) caused by metabolic changes triggered by diabetes is considered an essential factor in generating heart problems [6,7]. Oxidative stress during DM plays a crucial role in regulating coronary blood flow in response to myocardial metabolism (4) and is associated with a decrease in cardiac efficiency [8]. ROS, together with hyperglycemia, plays a central role in the initiation and progression of vascular damage, supporting the process of atherosclerosis and microvascular dysfunction [9]. Together, these conditions contribute to diabetic cardiomyopathy, which is closely associated with ischemic heart disease and heart failure [10]. The growing need to find alternatives for the treatment of diabetes justifies the study of medicinal plants used in traditional medicine.

Apocynin, a drug isolated from the medicinal herb Picrorhiza kurroa, is presumed to inhibit the expression of NADPH oxidase activity specifically and thus attenuate DM-induced oxidative stress in heart tissue [11]. Previous experiments have shown that apocynin can prevent endothelial dysfunction in diabetic rats, improved ventricular hypertrophy and fibrosis in a heart failure model of myocardial infarction [12,13]. Another study provided hemodynamic, biochemical, and molecular evidence that supported the therapeutic value of apocynin in improving adrenergic stress-induced cardiac hypertrophic [14]. Mitochondria that participate in the generation of reactive oxygen species (ROS) and apocynin have been involved in several disease models as aortic smooth muscle cell proliferation that contributes to the development of arterial remodeling and stenosis, hypertension, and atherosclerosis in metabolic syndrome [15], in hyperoxaluria induced nephrolithiasis [16], Parkinson’s disease [17], as well as in Helicobacter pylori-infected gastric epithelial cells [18]. The use of a novel mitochondrially targeted antioxidant, Mito-apocynin, has been used in cell culture models of neuroinflammation and mitochondrial dysfunction and an animal model of Parkinson’s disease showing enhanced mitochondrial function, anti-inflammatory properties, and attenuation of oxidative damage [19,20]. In this context, previous studies suggested an essential role of apocynin in the biochemical and molecular changes that occur in the heart. This study investigated apocynin’s influence as a beneficial treatment for reducing oxidative stress and improving antioxidant activity in heart tissue and mitochondrial functions with experimental diabetes.

## 2. Materials and Methods

### 2.1. Animals

Two-month-old male Wistar rats of 250 g were housed and maintained at 24 °C with 12-h day/night cycles, fed a standard rodent diet, and given water ad libitum. According to the Mexican Federal Regulations’ recommendations for the Use and Care of Animals (NOM-062-ZOO-1999, Ministry of Agriculture, Mexico), all experimental procedures took place. The Universidad Michoacana de San Nicolás de Hidalgo Institutional Committee approved the protocol performed.

### 2.2. Induction of Diabetes and Experimental Design

After 12 h of fasting, diabetes was induced in rats by intraperitoneal doses of streptozotocin (45 mg/kg of bodyweight) in 0.1 M citrate buffer (pH 4.5) as a vehicle, and normoglycemic rats were injected only with the vehicle. Twenty-four hours after streptozotocin injection, blood glucose levels were determined with an Accu-Check^®^ Performa glucometer (Roche, Indianapolis, IN, USA). Only the rats that exhibited fasting blood glucose of ≥300 mg/dL were employed for this study. After diabetes confirmation rats were randomly divided into 4 groups (*n* = 6): Control group (C), normoglycemic + apocynin group (A), diabetic group (D), and diabetic + apocynin group (D+A). Apocynin was administered daily at a dose of 3 mg/kg of bodyweight (vehicle, 0.1% dimethyl sulfoxide (DMSO) intraperitoneally for 5 weeks.

### 2.3. Metabolic Biomarkers

The animals were monitored for weight, basal glucose (fasting), and postprandial glucose using an Accu-Check^®^ Performa glucometer once a week during the 5 weeks of the experimental protocol.

### 2.4. Insulin Tolerance Test

After 5 weeks of treatment, an insulin tolerance test was performed. After 15 h of fasting, rats were administered a single dose of regular rapid-acting insulin (0.75 U/kg of bodyweight). Blood glucose levels were measured from baseline (before insulin administration t = 0 min); subsequently, blood glucose levels were measured at 30 min, 60 min, 90 min, and 120 min after insulin injection with an Accu-Check^®^ Performa glucometer. The glucose disappearance rate (KITT), derived from the insulin-tolerance test (ITT), was calculated by dividing 0.693 by the plasma glucose half-time (t1/2) × 100 as described previously [21,22]. The plasma glucose t1/2 was calculated from the slope of least square analysis of the glucose concentrations after i.p. insulin injection during the linear phase of decline. This test indicates the action of insulin in periphery tissues showing their sensitivity to the hormone [23].

### 2.5. Tissue Preparation

The next day after the insulin tolerance test, rats were sacrificed by decapitation and the heart was dissected and immediately placed in an ice-cold buffer (100 mM KCl, 40 mM Tris HCl, 10 mM Tris base, 5 mM MgCl2 y 1 mM EDTA, pH 7.4), chopped and homogenized, and stored at −80 °C until use. Protein content was routinely determined by the Biuret method with bovine serum albumin (BSA) as standard [24].

### 2.6. Determination of Catalase Enzyme Activity

Using a Clark-type oxygen electrode (5300A Biological Oxygen Monitor, YSI, Yellows Springs, OH, USA), catalase activity was assayed by measuring the conversion of H_2_O_2_ according to [25]. First, 0.5 mg of protein of heart homogenate were resuspended in a 50 mM potassium phosphate buffer (pH 7.6) at 25 °C, and the trace was monitored for 1 min. Next, the conversion of H_2_O_2_ to oxygen was recorded for 2 min by adding H_2_O_2_ to the chamber. Catalase activity was calculated using bovine catalase as standard (expressed as U/mg of protein).

### 2.7. Measurement of Reactive Oxygen Species

ROS levels were determined according to Ortiz-Avila et al. [26] by using the cell-permeable fluorescent probe 2′,7′-dichlorodihydrofluorescein diacetate (H_2_DCFDA). 0.5 mg of protein were placed in 2 mL of buffer containing 100 mM KCl, 10 mM HEPES, 3 mM KH_2_PO4, and 3 mM MgCl_2_ (pH 7.4) and incubated with 12.5 μM of H_2_DCFDA for 15 min in an ice-bath under constant shaking and the fluorescence was recorded at 0 and 60 min at 485 nm (ext) and 520 nm (em), in a spectrofluorophotometer (Shimadzu RF-5301PC, Kyoto, Japan) (expressed as units/mg of protein).

### 2.8. Heart Mitochondria Isolation

Heart mitochondria were isolated, as described by Saavedra-Molina and Devlin, with modifications [27]. In brief, tissue was chopped using a tissue-tearor homogenizer and incubated for 5 min with Trypsin/EDTA solution (2.5 mg/mL/0.2 mg/mL) in isolation media 1 (70 mM sucrose, 220 mM mannitol, 2 mM MOPS and 1 mM EGTA, pH 7.4). The reaction was stopped by adding a protease inhibitor cocktail (completeTM, Mini protease inhibitor cocktail) according to the manufacturer’s instructions and incubated for 1 min. Next, the samples were transferred to a Potter-Elvehjem-type glass homogenizer and homogenized with a Teflon pestle at 750 rpm in an ice bath. The homogenate was centrifuged at 2000 rpm for 10 min at 4 °C. Next, the supernatant was decanted and centrifuged at 7500 rpm for 10 min at 4 °C. The supernatant was removed, and the pellet resuspended carefully in isolation medium 2 (220 mM mannitol, 70 mM sucrose, 2 mM MOPS, and 0.2% *w/v* BSA) and centrifuged at 10,000 rpm for 10 min at 4 °C. Finally, the pellet was resuspended carefully in isolation medium 2 and stored at −80 °C until use.

### 2.9. Determination of the Enzymatic Activities of Complexes of the Electron Transport Chain

Complex I activity was determined spectrofluorometrically using the NADH autofluorescence according to [28]. Mitochondria from the different samples were solubilized in 3 repeated freeze/thaw cycles. First, 100 μg/mL of solubilized mitochondria were resuspended in potassium phosphate buffer (50 mM, pH 7.4) and incubated for 5 min with 1 μg antimycin A and 0.5 mM KCN. Next, 40 μL of 10 mM K3[Fe(CN)6] was added to the mixture, and fluorescence was recorded at excitation/emission wavelengths 352/464 nm in a Shimadzu RF-5301PC. After 1 min, 100 μM β-NADH (β-nicotinamide adenine dinucleotide reduced) was added, and the fluorescence was monitored for 2 min, then 5 μM rotenone was added to inhibit the reaction, and the fluorescence was monitored for 2 min. For Complexes II, II + III, and IV activity determination, mitochondria from the different samples were solubilized in 3 repeated freeze/thaw cycles followed by osmotic shock, according to Spinazzi [29] with slight adaptations. Complex II activity, 0.2 mg of mitochondria were resuspended in deionized water and incubated for 2 min to induce an osmotic shock. Next, potassium phosphate buffer (250 mM, pH 7.5) was added to a final concentration of 50 mM, followed by the addition of 5 mM succinate, 5 μM of rotenone, 1 μg antimycin A, 0.5 mM KCN and 0.1 mg of BSA, mixed and incubated for 3 min. Then, 80 μM DCIP was added, and the absorbance changes were monitored for 2 min at 600 nm; then, 0.5 mM of TTFA was added to inhibit the reaction, and the absorbance was monitored for 2 min [30]. Complexes II–III activity, 0.2 mg of mitochondria were resuspended in deionized water as previously described. Thereupon, potassium phosphate buffer (250 mM, pH 7.5) was added to a final concentration of 50 mM and incubated 3 min with 5 mM succinate, 5 μM of rotenone, and 0.5 mM KCN and 0.1 mg of BSA. Later, 250 μg cytochrome *c* was added, and the changes in absorbance were monitored for 2 min at 550 nm, and 1 μg antimycin A was added and monitored for 2 min [31]. Complex IV activity, 0.1 mg of mitochondria were resuspended in deionized water as previously described. Next, potassium phosphate buffer (250 mM, pH 7.5) was added to a final concentration of 50 mM and mixed with 5 μM of rotenone, 0.5 mM TTFA, 1 μg antimycin A and 0.1 mg of BSA, and incubated 3 min. Then, 125 μg cytochrome *c* reduced (by sodium dithionite) were added and the absorbance was monitored for 1 min at 550 nm. Finally, 0.5 mM KCN was added and monitored for 1 min [32]. All experiments were performed at 30 °C in a spectrometer Perkin-Elmer UV/Vis Lambda 18.

### 2.10. Determination of Glutathione Peroxidase Activity

According to the method described by Lawrence and Burk with modifications, the GSH-Px activity was determined [32]. 0.2 mg of mitochondria were resuspended in potassium phosphate buffer 50 mM and 5 mM Na_2_EDTA and mixed with 1 mM glutathione reduced 1 mM NaN_3_, 0.1 mg BSA, and 100 mU/mL of glutathione reductase and incubated 5 min. Next, 100 μM of NADPH (β-nicotinamide adenine dinucleotide 2′-phosphate reduced) was added and incubated for an additional 1 min, and the fluorescence was monitored 1 min at excitation/emission wavelengths 352/464 nm, then 250 μM H_2_O_2_ was added, and the changes in fluorescence were monitored 3 min at 30 °C in a Shimadzu RF-5301PC. A separate reaction blank was prepared by replacing the sample with deionized water and subtracted from each assay.

### 2.11. Determination of Glutathione Status

Total glutathione, GSSG and GSH, were determined using an enzymatic recycling method according to [28]. In brief, 100 μL of mitochondria were resuspended in a blend containing 0.1% Triton-X, 0.6% sulfosalicylic acid, 5 mM Na_2_EDTA in 50 mM potassium phosphate buffer. The samples were sonicated in 3 cycles (sonication/ice) in a Branson sonifier 450 with a tapered microtip (20W output/constant duty cycle); next, the samples were subject to 2 repeated freeze/thaw cycles and centrifugated for 10 min at 7200 rpm. Later, 100 μL of the supernatant was placed in a mixture containing 5 mM Na_2_EDTA, 0.1 mM 5,5′-dithiobis-2-nitrobenzoic acid and 100 mU/mL glutathione reductase in 50 mM potassium phosphate buffer and incubated for 1 min at room temperature. Then, the reaction was started by the addition of 50 μM β-NADPH and the change in absorbance was registered at 412 nm at 30 °C in a spectrometer Perkin-Elmer UV/Vis Lambda 18 and changes in absorbance were recorded for 5 min. GSSG from the different samples was determined after incubation with 0.2% 4-vinylpyridine at room temperature for 1 h. GSH from the different samples was calculated by subtracting GSSG from the total glutathione and the GSH/GSSG ratio was calculated by dividing the GSH levels by the GSSG levels.

### 2.12. Determination of Superoxide Dismutase

The enzymatic activity of superoxide dismutase (SOD) was determined using Sigma-Aldrich SOD assay kit-WST (19160) (Sigma, St. Louis, MO, USA), following the manufacturer’s instructions.

### 2.13. Statistical Analysis

Results were expressed as the mean ± standard error. Statistical analyses were performed with one-way or two-way analysis of variance (ANOVA) with Tukey’s multiple comparison test. A *p*-value of ≤ 0.05 was considered statistically significant. All data were analyzed using Prism (GraphPad 7.0 version, Inc., San Diego, CA, USA).

## 3. Results

### 3.1. Effect of Apocynin on Bodyweight and Fasting and Postprandial Blood Glucose Levels

**Figure 1:**
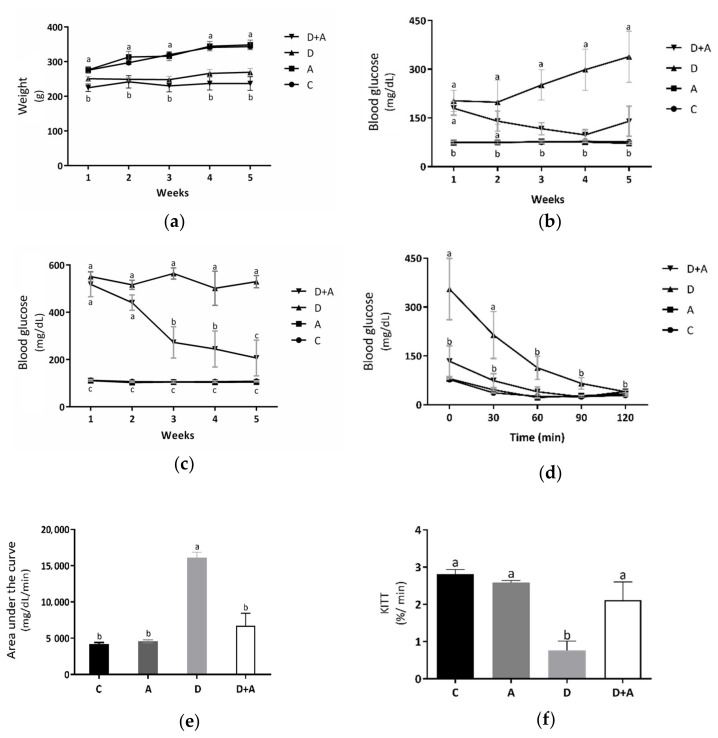
Effect of Apocynin on metabolic biomarkers. This figure shows bodyweight of the different groups of rats (**a**) (g); fasting glucose levels (**b**) (mg/dL); postprandrial glucose levels (**c**) (mg/dL); glucose levels in the insulin-tolerance test (ITT) (**d**) (mg/dL); area under the curve (**e**) (mg/dL/min); glucose disappearance rate (KITT) during the ITT (**f**) (%/min). C = control; A = apocynin; D = diabetic; D+A = diabetic + apocynin. *n* = 6. Data are presented as the mean ± standard error. (2-way ANOVA, Tukey post-hoc test). The different letters (lower case) indicate the significant differences between groups with *p* < 0.05.

Metabolic biomarkers such as bodyweight (Figure 1a and fasting (Figure 1b and postprandial blood glucose levels (Figure 1c were evaluated to determine the effect of apocynin in the different groups. Measurements were made during the treatment period (5 weeks). As shown in Figure 1a, it was observed that the results obtained from the bodyweight measurements did not show significant differences in the group treated with apocynin (319.7 ± 11.71 g) compared to the control group (315.4 ± 4.56 g). In the diabetic rats treated with apocynin (233.49 ± 13.29 g), a reduction in bodyweight was observed compared to the control group of 26%. However, there were no significant differences with the diabetic group. It indicated that apocynin does not affect the bodyweight of diabetic rats. In fasting blood glucose levels (Figure 1b, the group treated with apocynin (74.53 ± 2.56 mg/dL), compared to the control group (76.06 ± 2.09 mg/dL), there were no significant differences, while in the diabetic group (258.1 ± 52.29 mg/dL) there were increased values by 239% compared to the control group. The diabetic group treated with apocynin (134.93 ± 10.21 mg/dL) presented glucose levels increased by 77%, concerning the control group, but did not present statistically significant differences. As of the third week, significant differences between the diabetic group and the diabetic group treated with apocynin began to be observed, demonstrating that the drug affects the blood glucose levels of diabetic rats. Figure 1c shows the postprandial blood glucose levels. There were no significant differences between the control group (107.8 ± 2.58 mg/dL) and the group treated with apocynin (102.03 ± 2.93 mg/dL). On the other side, in the diabetic group (532.46 ± 27.64 mg/dL), an increase of 394% was observed, compared to the control group, and there were significant differences. The diabetic group treated with apocynin (336.53 ± 49.62 mg/dL) presented glucose levels increased by 212%, compared to the control group. What can be observed in the graph is that both diabetic groups (treated and untreated), in the first two weeks, had significant differences against the other two groups, but not between each other. However, in week three, in the diabetic group treated with apocynin, blood glucose levels began to decline gradually. A 47% decrement was observed in the first week, 53% in the third week, and 60% in the fourth. There was no longer a statistically significant difference between this group and the control in the fifth week. Taking this into account, what was described in the previous figure was corroborated; the treatment from the third week began to have positive effects on glucose levels, either in fasting or postprandial glucose of rats with DM. The insulin sensitivity test that can be observed in Figure 1d showed a significant increase in blood glucose in diabetic rats, compared to the control group as follows: 355.2 ± 94.17 mg/dL at 30 min, 214.14 ± 72.07 mg/dL at 60 min, and 76 ± 4.08 mg/dL at 30 min, 37.5 ± 1.43 mg/dL at 60 min, respectively. After 90 min, the diabetic group did not show significant differences against the control group, indicating a deterioration in glucose uptake during the first hour, a situation established in experimental diabetes, but this became normal during the following periods. On the other side, in the group of diabetic rats + apocynin, it was observed that from the beginning, their blood glucose values (133.8 ± 47.64 mg/dL at baseline, 74 ± 21.35 mg/dL at 30 min, 39.8 ± 14.79 mg/dL at 60 min, 24.2 ± 0.91 mg/dL, at 90 min and 40.8 ± 3.77 mg/dL at 120 min) did not present statistically significant differences compared with the control groups. With the results obtained from the blood glucose during the insulin sensitivity test, the area under the curve (AUC) (Figure 1e was calculated as an integrated expression of the glucose concentration. The AUC increased significantly in the diabetic group (16,132 ± 742.14 mg/dL/min) compared to the control group (4218.6 ± 182.42 mg/dL/min) by 282%; this, as a function of the significantly higher values of glucose in the blood that was present during the pathology. In contrast, the group treated with apocynin (6758.2 ± 1510.53 mg/dL/min) increased by only 60% concerning the control but did not show significant differences. Comparing the diabetic + apocynin group against the diabetic without treatment, a difference of 222% was appreciated, with significant values of a *p* < 0.0001, thus showing that the drug had positive effects on insulin sensitivity to lower blood glucose levels. As depicted in Figure 1f, the KITT values, an index of insulin sensitivity, were significantly lower in the diabetic group (0.77 ± 0.24%/min; *p* = 0.001) than in the control group (2.80 ± 0.13%/min). In contrast, the KITT value of the diabetic+apocynin group (2.11 ± 0.48%/min; *p* = 0.04) was significantly higher compared with the untreated diabetic group, thus showing that the drug had positive effects on insulin sensitivity.

### 3.2. Effect of Apocynin on the Levels of Reactive Oxygen Species in the Heart Muscle

**Figure 2:**
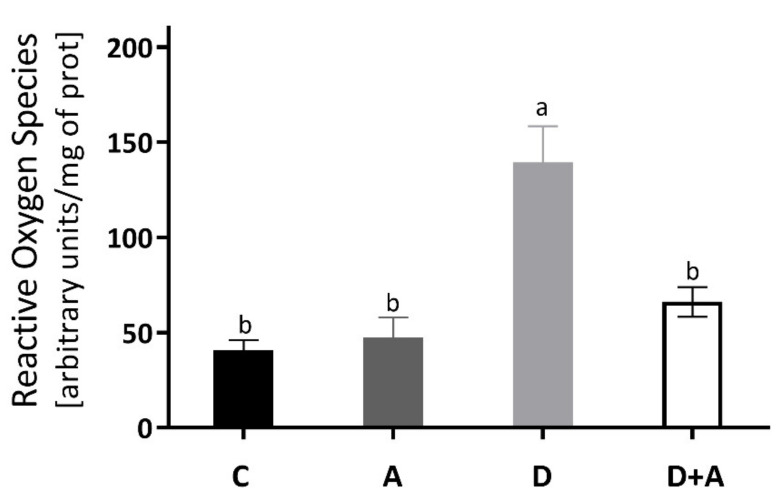
Levels of Reactive Oxygen Species in the heart of different groups of rats. This figure shows the Reactive Oxygen Species (ΔF) levels in the heart for each group C = control, A = apocynin, D = diabetic, D+A = diabetic + apocynin. *n* = 6; Data are presented as the mean ± standard error (One-way ANOVA, Tukey posthoc test). The different letters indicate the significant differences between groups, with *p* < 0.05.

The results obtained for the determination of ROS of the heart muscle are shown in Figure 2, and it was observed that there was an increase of 244% in the group of diabetic rats (140.10 ± 16.009 ΔF) compared to the control group (40.68 ± 5.45 ΔF). Even though the diabetic group + apocynin (65.33 ± 4.60 ΔF) had an increase of 61% in ROS levels, it did not present significant differences to the control group, nor the control group treated with apocynin (47.40 ± 10.64 ΔF). On the other hand, when comparing the diabetic group to the diabetic group with treatment, a significant difference of ** *p* < 0.0016 was observed (an increase of 180.20%).

### 3.3. Effect of Apocynin on Catalase Activity in the Heart Muscle

**Figure 3:**
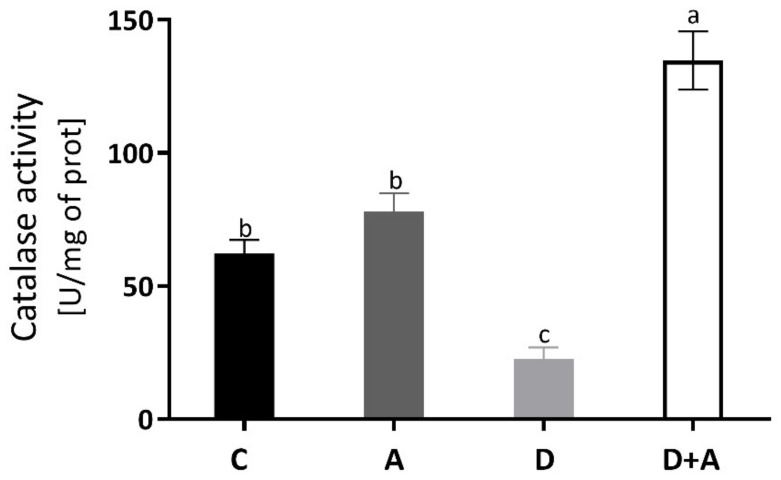
Catalase enzyme activity in the heart of different groups. This figure shows the catalase enzyme activity (U of activity/mg of prot) in the heart for each group C = control, A = apocynin, D = diabetic, D+A = diabetic + apocynin. *n* = 6. Data are presented as the mean ± standard error (One-way ANOVA, Tukey posthoc test). The different letters indicate the significant differences between groups, with *p* < 0.05.

The catalase enzyme activity was measured to evaluate the antioxidant system, and the results are observed in Figure 3. In the diabetic group (22.72 ± 9.32 U/mg of prot) without treatment, a decrease of 63% was observed compared to the control group (62.13 ± 11.66 U/mg of prot), presenting statistically significant differences. In the diabetic group + apocynin (134.69 ± 24.44 U/mg of prot), there was an increase of 117% compared to the control group; significant differences were presented. The control group + apocynin (78.03 ± 15.51 U/mg of prot) presented an increase of 26%, without being statistically significant compared to the control group. The diabetic group and the diabetic group treated with apocynin did.

### 3.4. Effects of Apocynin on Mitochondrial Complex Activities

**Figure 4:**
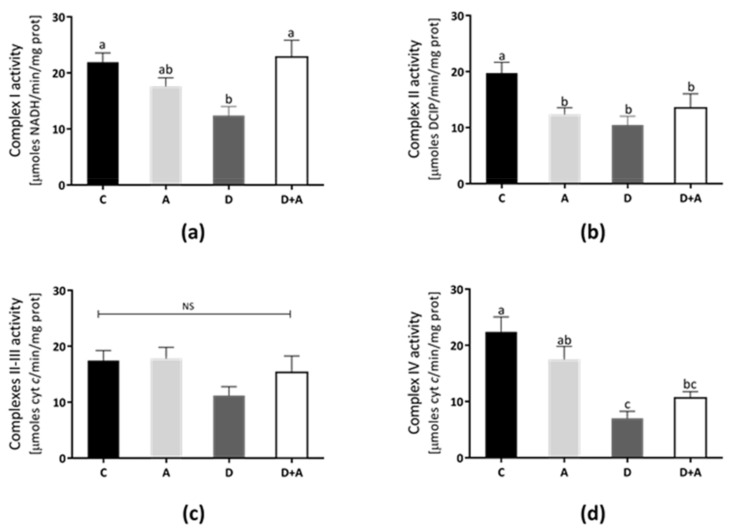
Effect of apocynin on enzymatic activities of complexes of the electron transport chain (ETC) in heart mitochondria. (**a**) Complex I activity. (**b**) Complex II activity. (**c**) Complexes II–III. (**d**) Complex IV. Different letters indicate significant differences between groups, ns: Not significant, (*p* ≤ 0.05) by one-way ANOVA with Tukey post hoc test. Values represent means ± SEM. Control group, C, normoglycemic + apocynin group, A, diabetic group, D, and diabetic + apocynin group, D+A.

The rat heart mitochondrial complexes’ activities were determined to elucidate the apocynin effects on mitochondrial functions (Figure 4). In Complex I (Figure 4a), Complex II (Figure 4b), Complex III (Figure 4c), and Complex IV (Figure 4d) show that the lowest activity values were in the diabetic group (40%, 36%, 29%, and 70%, respectively). In contrast, Complex I and II/III activities from the apocynin + diabetes group (D+A) were normalized, compared to control (10% above control, and were significantly increased compared with the diabetic group, for Complex I only). On the other hand, in the apocynin group, Complex III was increased 6% above control (Figure 4c), whereas, in the apocynin + diabetes group, the activity increased to 24% compared diabetic group. Complex IV activity increased by 42% in the apocynin + diabetes group compared diabetic group (Figure 4d).

### 3.5. Effects of Apocynin on Oxidative Stress in Heart Mitochondria

**Figure 5:**
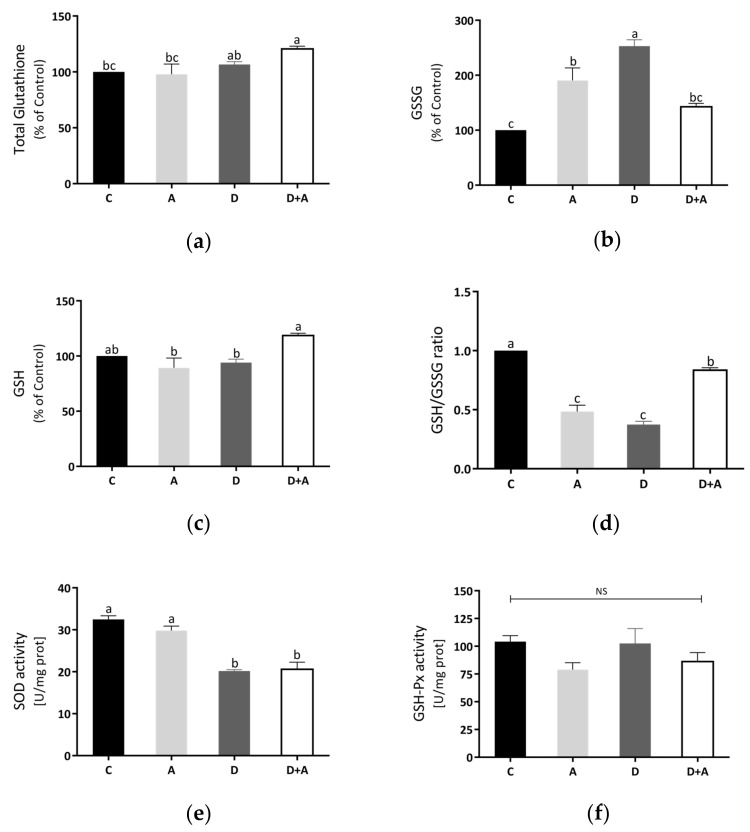
Effect of apocynin on oxidative stress in heart mitochondria. (**a**) Total glutathione. (**b**) Oxidized glutathione. (**c**) Reduced glutathione. (**d**) GSH/GSSG ratio. (**e**) SOD activity. (**f**) GSH-Px activity. Different letters indicate significant differences between groups; ns: Not significant (*p* ≤ 0.05) by one-way ANOVA with Tukey post hoc test. Values represent means ± SEM. Control group, C, normoglycemic + apocynin group, A, diabetic group, D, and diabetic + apocynin group, D+A.

Total glutathione and GSH were increased in the diabetes + apocynin group in Figure 5a,c, compared to the control group. The oxidized GSSG values had a significant increase in the diabetes group, although a diminution was observed in the diabetes + apocynin group (Figure 5b). The GSH/GSSG ratio was significantly diminished in the diabetes group, with a significant increase in the diabetes + apocynin group (Figure 5d). We also analyzed the antioxidant mitochondrial biomarker enzymes, superoxide dismutase 2, and glutathione peroxidase (Figure 5e,f). SOD2 activity was diminished in the diabetes group and showed a significant increase in the apocynin group (Figure 5e). At the same time, GSH-Px activity showed no significant differences between the different groups tested (Figure 5f).

## 4. Discussion

The chronic hyperglycemic state characteristic of DM is linked to oxidative stress damage in various tissues, a critical component involved in triggering diabetic complications, such as diabetic cardiomyopathy [7]. Thus, ROS has emerged as a target for therapy against diabetes, using based strategies to boost cellular antioxidant capacity and ROS detoxification to mitigate oxidative stress. In the current study, we have shown the effects of apocynin administration during five weeks as a beneficial treatment by reducing oxidative stress, lowering blood glucose levels, increasing insulin sensibility, leading to improve cellular and mitochondrial-localized antioxidant defenses in heart tissue of STZ-induced diabetic rats. Our findings also identify the glutathione status redox and mitochondrial ETC as apocynin’s targets in diabetic cardiac mitochondria.

Consistently, our study demonstrates that in the diabetic group, metabolic parameters were significantly affected; fasting and postprandial hyperglycemia, low insulin sensitivity, as well as a noticeable lower weight, compared to the control group (Figure 1), which matches what was previously reported in rats treated with STZ to establish an experimental diabetes model [3,4]. Notably, apocynin treatment at a dose of 3 mg/kg contributed to a significant reduction of fasting and postprandial glucose levels from the third week of treatment; however, no differences were observed in the bodyweight gain of the diabetic group (Figure 1). In support of our results, a previous report revealed that inhibition of renal gluconeogenesis is involved in apocynin hypoglycemic action in diabetic rabbits [33]. Furthermore, insulin sensitivity significantly improved after apocynin treatment in diabetic rats (Figure 1e), which is also similar to other authors’ results, where following apocynin treatment for five weeks markedly, there was an improvement in insulin sensitivity in high-fat diet (HFD)-induced obese mice [34]. Subsequently, similar findings demonstrated that apocynin significantly reduced hyperglycemia, hyperinsulinemia, and dyslipidemia by improving insulin sensitivity in HFD fed mice as well [35].

Diabetes-related systemic insulin resistance causes alterations in cardiac metabolism, and hyperglycemia undoubtedly elicits detrimental cardiomyocyte function effects [36]. The loss in flexibility between energy sources causes reduced cardiac efficiency, contractile dysfunction, with adverse tissue remodeling, which is a hallmark of diabetic cardiomyopathy. Moreover, there is evidence linking a disrupted cardiac insulin signaling to overproduction of ROS, mitochondrial dysfunction, and oxidative stress, which are significant metabolic abnormalities implicated as well [36,37]. In our study, STZ-induced insulin deficiency triggered the overproduction of ROS in the diabetic heart. Meanwhile, apocynin significantly reduced ROS levels compared to the diabetic group (Figure 2). Increased ROS levels in diabetic hearts and high glucose-incubated cultured cardiomyocytes have been mostly attributed to NADPH oxidase activity [38,39].

Apocynin has gained importance as an antioxidant agent in experimental research. Evidence suggests that NADPH oxidase inhibition with apocynin attenuates isoproterenol-induced myocardial damage by enhancing antioxidant status [40]. This fact coincides with Gimenes et al. [1], where they indicated that apocynin restores serum antioxidant enzyme activities catalase and SOD in diabetic rats. Likewise, our results agreed by showing that chronic inhibition of the NADPH-oxidase by apocynin restoring catalase antioxidant enzyme activity in diabetic hearts (Figure 3), which is a crucial component of the intracellular ROS detoxification system. Properties of apocynin as ROS scavenging and antioxidant have been previously reported in diabetic complications such as endothelial dysfunction [41], nephropathy [42], retinopathy [43], and diabetic mitochondrial dysfunction in skeletal muscle [44]. In addition, there are reports associated with the amelioration of adverse cardiac effects in other pathological conditions [45,46].

On the other hand, studies of mitochondrial function and morphology in the heart support a connection between mitochondrial dysfunction and DM [47,48]. Our data consistently showed changes in mitochondrial function, evidenced by a significant reduction in ETC complexes’ activity in diabetic rats (Figure 4). The decreased respiratory complexes’ activities may increase electron leak, generating more superoxide anion radical and subsequent overproduction ROS that may cause damage to specific complex subunits and contribute to mitochondrial dysfunction [47,49]. Therefore, these results can represent the consequent mitochondrial dysfunction, mainly by decreasing Complex IV resulting in lower ATP production, increased mitochondrial ROS, and oxidative damage [50]. Mitochondrial oxidative stress was evident by increased GSSG levels and reduced GSH/GSSG ratio; concomitantly, we observed reduced activity of mitochondrial SOD in heart mitochondria from diabetic rats (Figure 5). Modifications due to oxidative damage in the diabetic heart’s mitochondrial complexes may play a key role in the gradual decline in mitochondrial function [48].

Moreover, decreased GSH and increased GSSG levels during oxidative stress can contribute to mitochondrial dysfunction by glutathionylation of target proteins [51]. However, we found that the decrease in Complexes I and IV detected in diabetic rats was significantly prevented by apocynin treatment, but only the Complex I and III activities were found to be similar to those detected in control rats. Notwithstanding, apocynin did not significantly influence the Complex II activity. In contrast, apocynin treatment in the control group led to a decrease in Complex II activity. A possible explanation for this effect can be due to a depletion of the coenzyme Q pool that could directly impair oxygen consumption rate, as has been suggested [52], or even by reacting apocynin with Complex II (succinate dehydrogenase) that contains two hydrophilic subunits, three Fe-S centers, a flavoprotein, and two hydrophobic subunits, which contain one heme b and the binding site for Q [53]. Additionally, a diminution in the cellular antioxidant systems has been reported when GSH negatively affected enzymes as succinate dehydrogenase (Complex II), while exogenous administration of glutathione prevented oxidative stress and a loss of enzymatic activities affected by oxidative modifications [54,55]. In this context, we hypothesize the diminution of succinate dehydrogenase activity could be due to the loss of redox balance caused by apocynin, as we observed in our results.

Based on other research, apocynin’s beneficial properties to improve mitochondrial function are decreasing the level of NADPH oxidase activity, mitochondrial DNA oxidative damage, and increasing the level of SOD activity, which suggests that apocynin efficiently decreases NADPH oxidase-associated oxidative stress [56]. In line with this, studies using hearts from diabetic animals showed that upregulation of NADPH oxidase by activation of CaMKII might lead to morphological changes of mitochondria [57]. Therefore, in addition to mitochondria, NADPH oxidase may be the primary ROS source in the diabetic heart [48]. In turn, the redox imbalance on glutathione status in mitochondria from diabetic rats was mitigated by apocynin, confirming its antioxidative action manifested by a significant increase in GSH/GSSG ratio. In addition, the changes observed in Figure 5 could be attributed to a direct interaction of apocynin with GSH or thiol groups and increased levels of GSSH [58].

Similarly, apocynin has turned out to produce a beneficial effect on glutathione homeostasis in various mammalian tissues, including in kidneys of the Zucker diabetic fatty (ZDF) rat [59] and spontaneously hypertensive Dahl salt-sensitive rats [60], liver of high-fat diet-induced obese mice [35]. However, the present study is the first to show apocynin’s effects on glutathione redox status in mitochondria from STZ-diabetic rats’ hearts. Hence, these results suggest other possible mechanisms by which apocynin could mediate its cardioprotective effects against DM-induced oxidative stress, at least partly through its mitochondrial action acting as a mitochondria-targeted antioxidant and modulating respiratory chain enzyme activities.

## 5. Conclusions

In summary, apocynin enhances glucose uptake and insulin sensitivity, thereby preserving the antioxidant defense and mitochondrial function in diabetic rat hearts. These findings provide new insights into the mechanisms of apocynin antioxidative action. This study is the first showing the effects of apocynin on glutathione redox status and components of ETC in heart mitochondria from STZ-induced diabetic rats. Apocynin has been demonstrated as an efficient and antidiabetic drug in muscle heart in an experimental animal model of DM type 1.

## Data Availability

All data are included in the document.
